# Supplementary figures and images for: SLICER: inferring branched, nonlinear cellular trajectories from single cell RNA-seq data
Source: Genome Biol. 2016 May 23;17:106. doi: 10.1186/s13059-016-0975-3 (PMC4877799; doi:10.1186/s13059-016-0975-3)

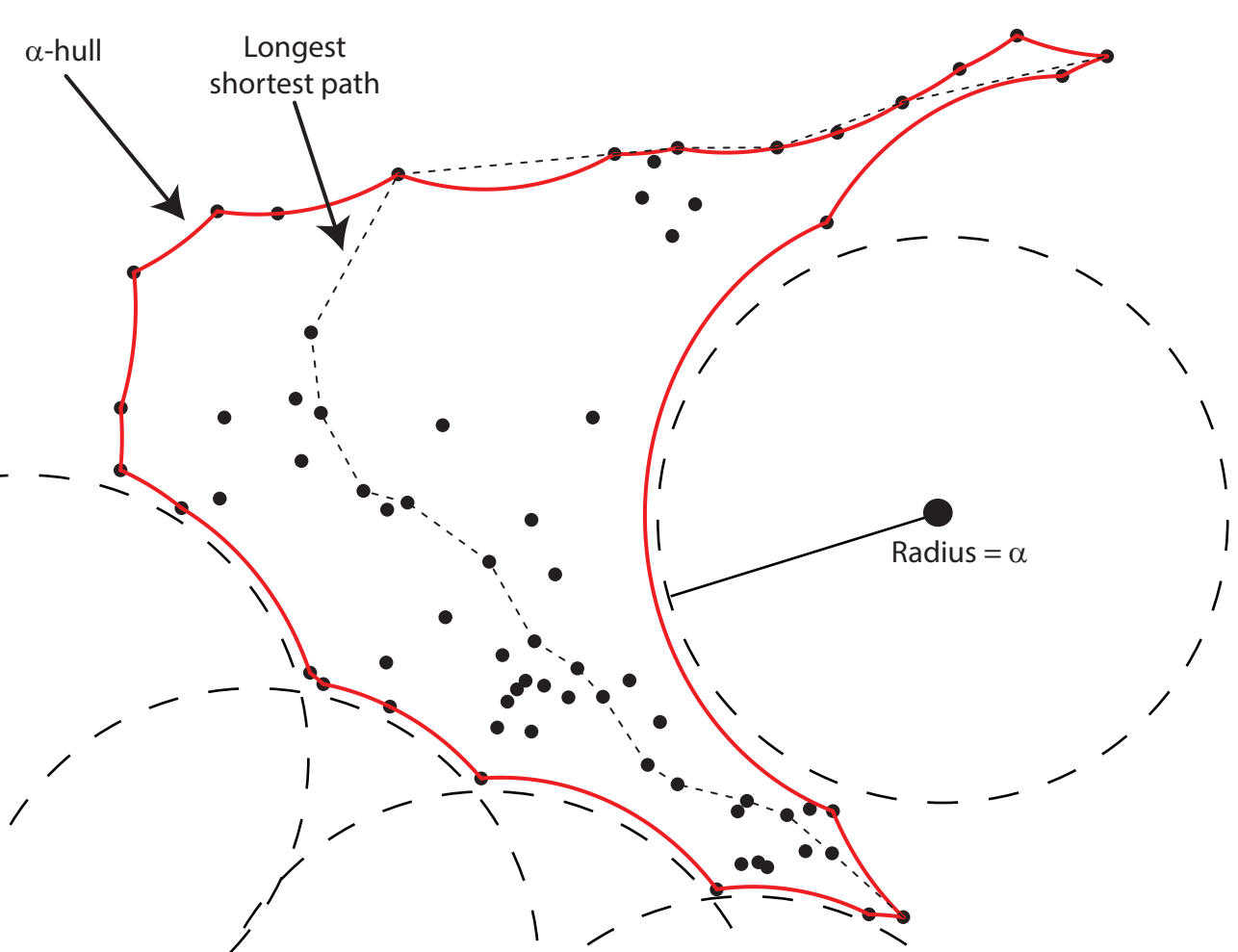

Supplement: Additional file 1: Figure S1. — Selecting k using the a-convex hull. The red border is the alpha-convex hull of the set of points shown, obtained by taking the intersection of the spheres of radius a indicated here. The longest shortest path is shown as a dotted line. (PDF 85 kb) [file 13059_2016_975_MOESM1_ESM.pdf]

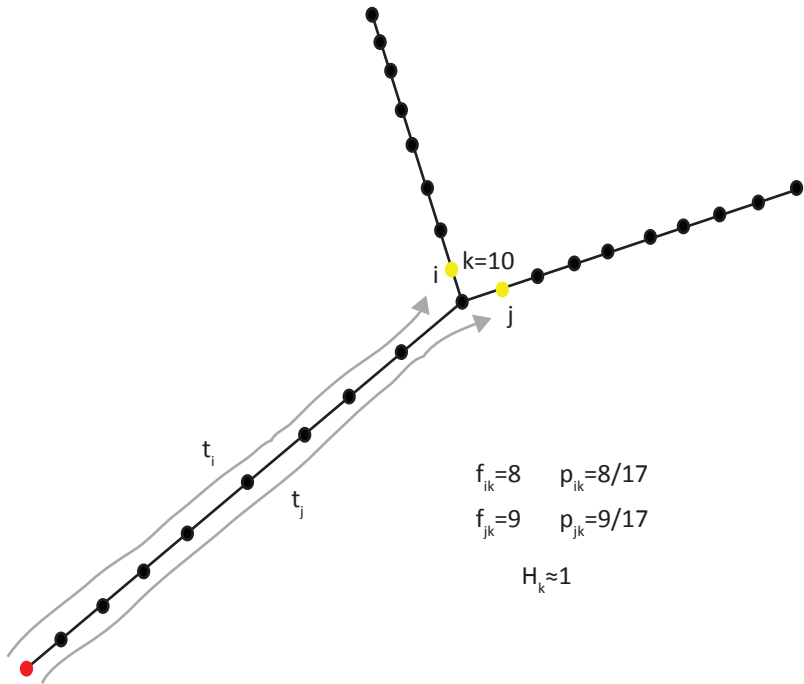

Supplement: Additional file 2: Figure S2. — Computing geodesic entropy of a trajectory. The starting cell is indicated in red, two geodesics (shortest paths) are shown in gray, and the cells at k = 10 steps away from the starting cell are indicated in yellow. (PDF 89 kb) [file 13059_2016_975_MOESM2_ESM.pdf]

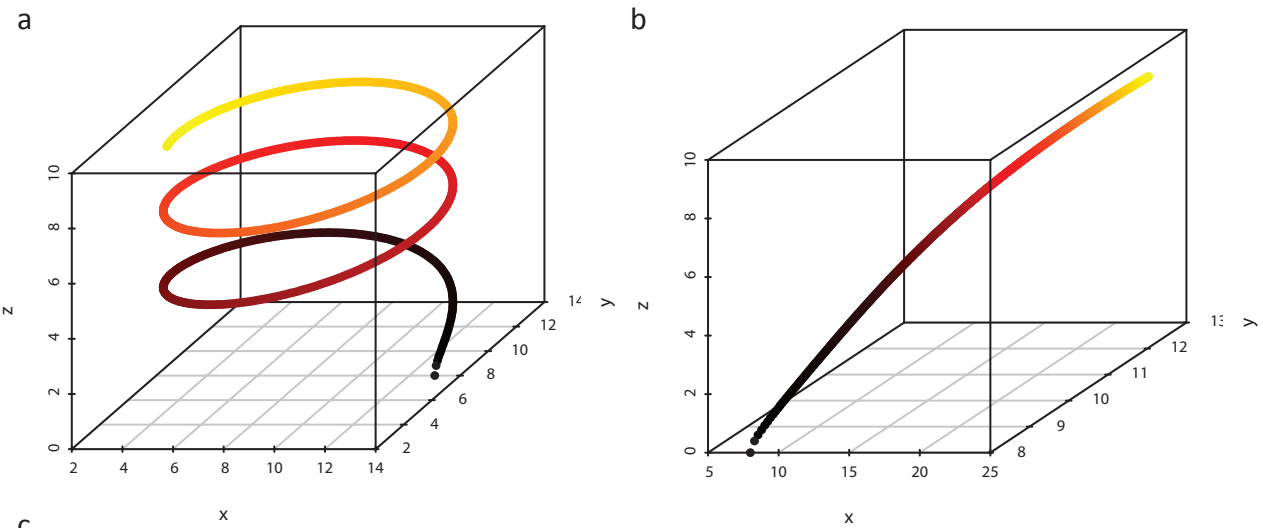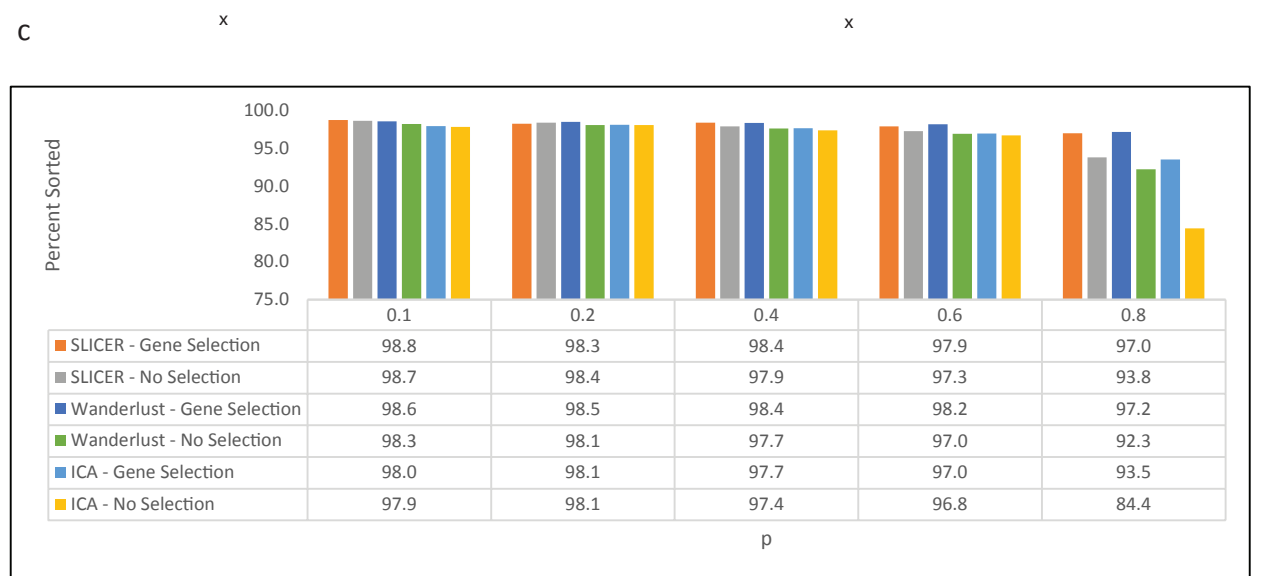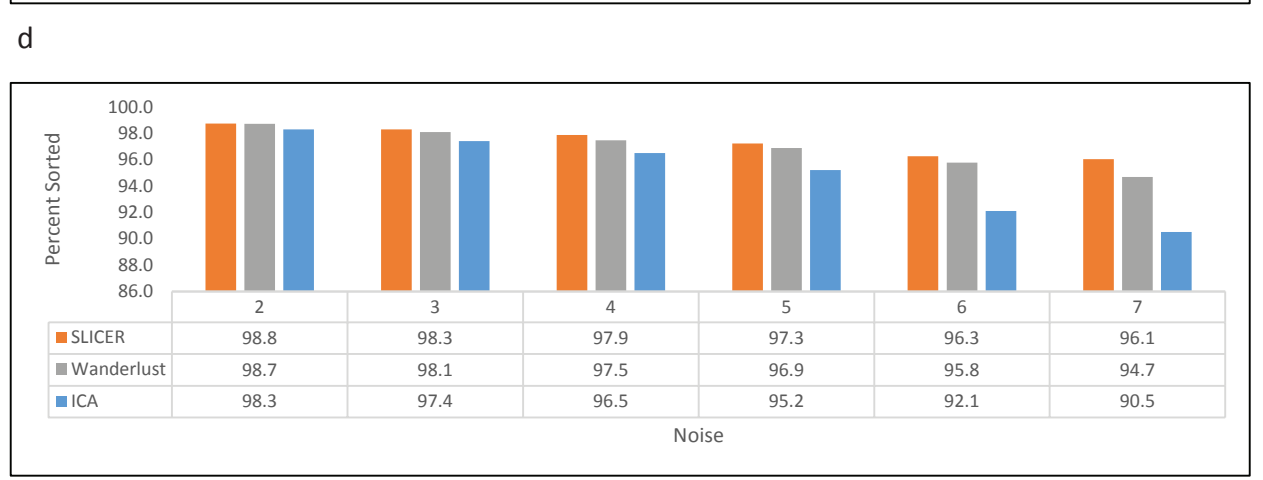

Supplement: Additional file 3: Figure S3. — Additional simulations comparing SLICER with other approaches. (a) The first three functions used to generate the synthetic data discussed in Fig. 2. Note the highly curved shape of the trajectory. (b) The first three functions used to generate an additional dataset. This trajectory is much less curved than the one shown in panel (a), and ICA thus performs much better on this example. (c) Performance of SLICER and other approaches, with and without gene selection, on the trajectory shown in panel (b) as the proportion of irrelevant genes increases. Note that the other approaches do not perform gene selection on their own, so the genes selected by SLICER were given as input for this comparison. A noise level of 2 was used for these simulations. Note that the y-axis does not start at 0. (d) Performance of SLICER and other approaches on the trajectory shown in panel (b) as the noise level increases. To isolate the effect of increasing noise, an irrelevant gene proportion of p = 0 was used for these datasets. Note that the y-axis does not start at 0. (PDF 146 kb) [file 13059_2016_975_MOESM3_ESM.pdf]

a

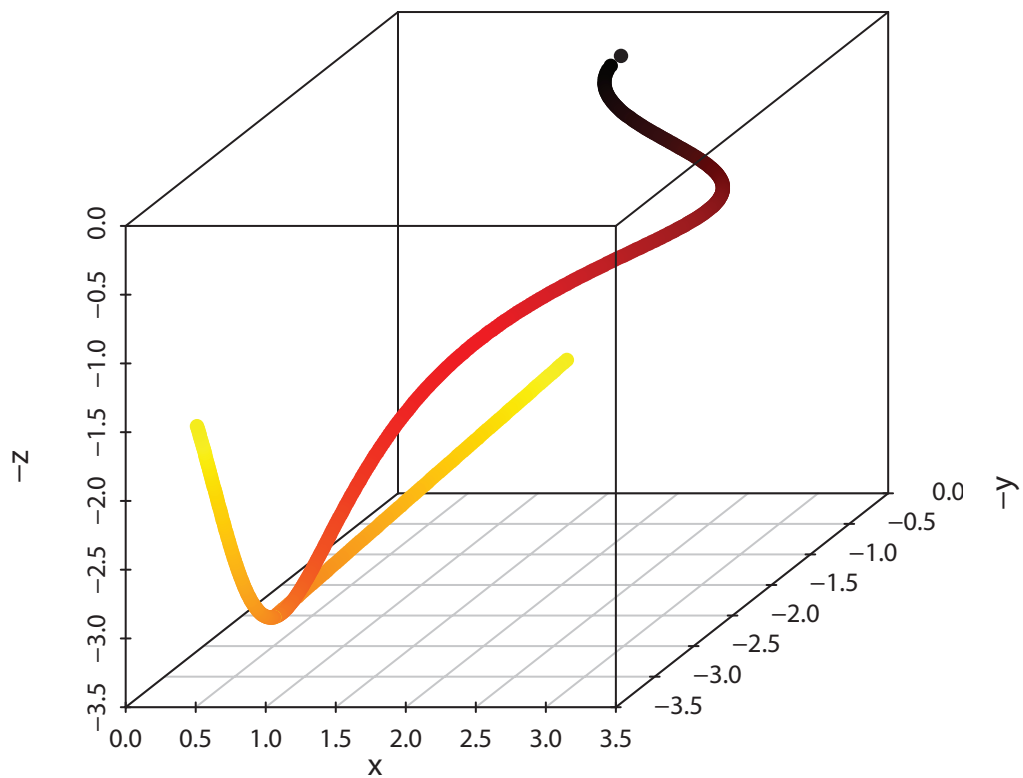

b

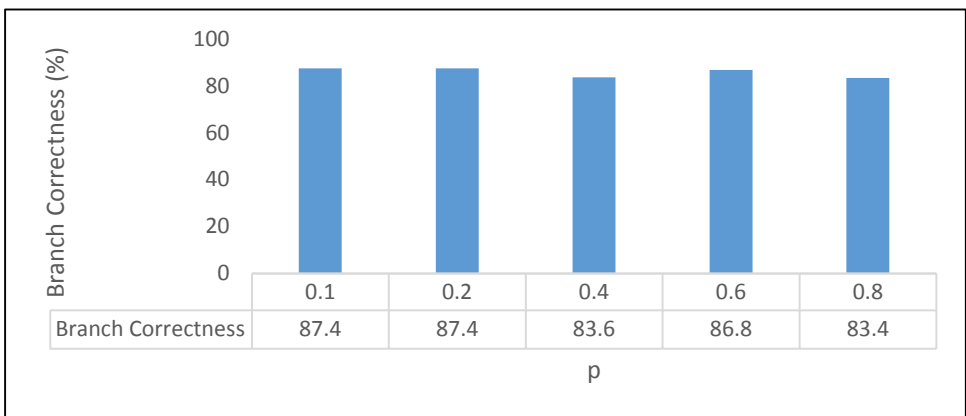

c

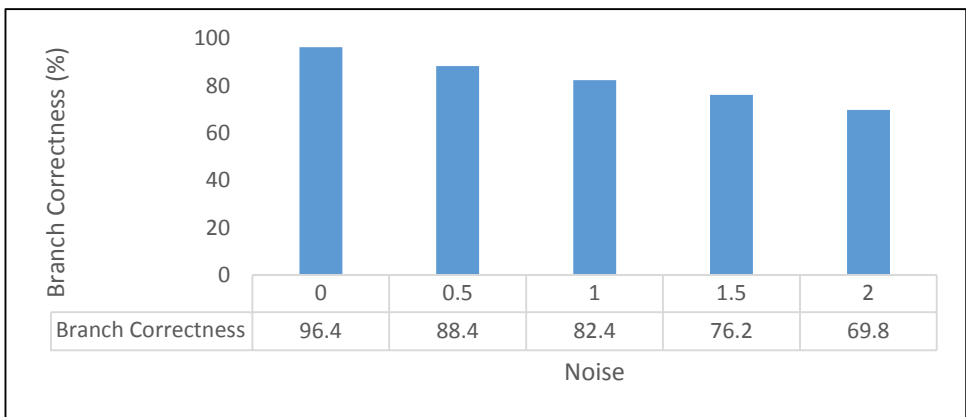

Supplement: Additional file 4: Figure S4. — Robustness of branch detection in the presence of noise and irrelevant genes. (a) Simulated branching trajectory used to assess the robustness of SLICER’s branch detection method. (b) Chart showing the percentage of cells assigned to the correct branch by SLICER as the proportion of irrelevant genes increases (noise = 0.5). (c) Percentage of cells assigned to the correct branch in the presence of increasing noise (p = 0). (PDF 98 kb) [file 13059_2016_975_MOESM4_ESM.pdf]

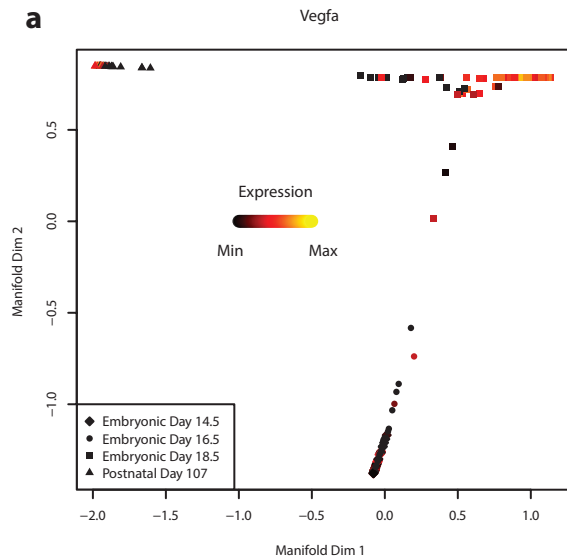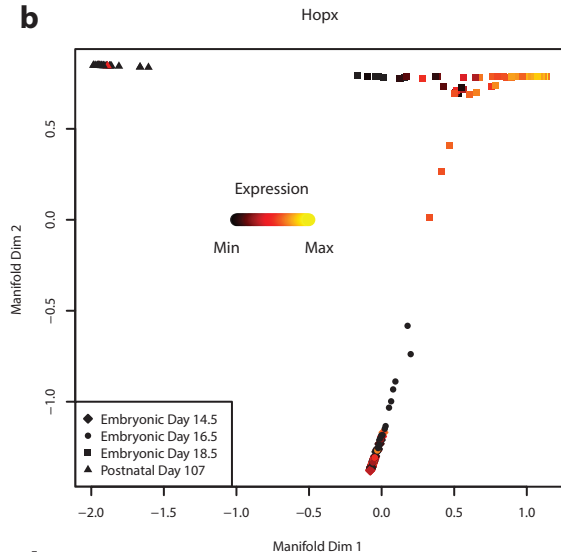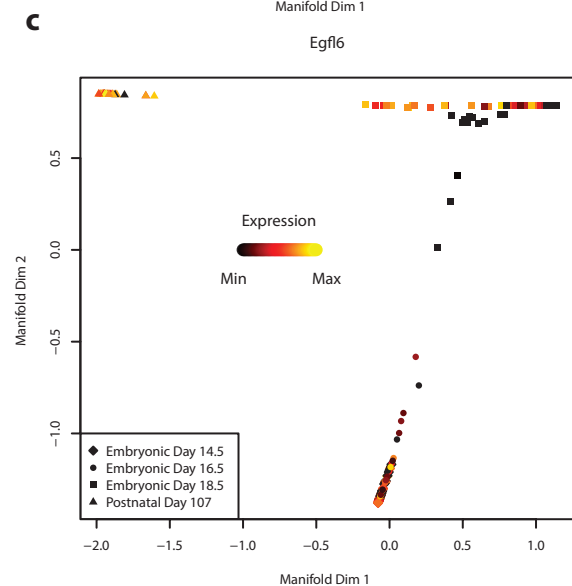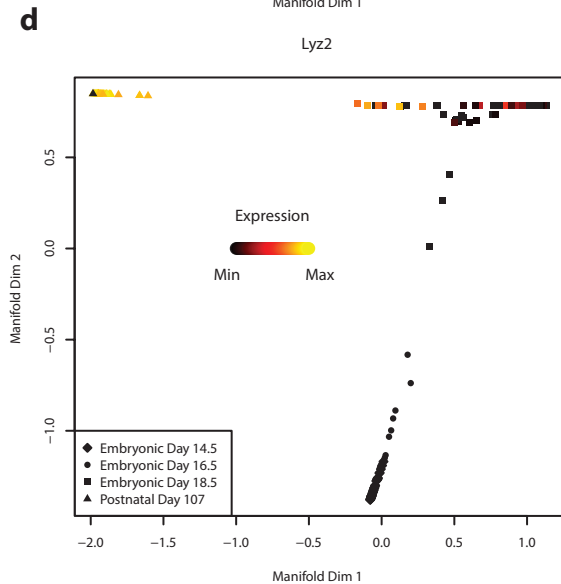

Supplement: Additional file 5: Figure S5. — Additional marker genes for mouse lung dataset. (a) and (b) are markers for alveolar type 1 (AT1) cells. (c) and (d) are markers for AT2 cells. (PDF 92 kb) [file 13059_2016_975_MOESM5_ESM.pdf]

**a**

Cd24a

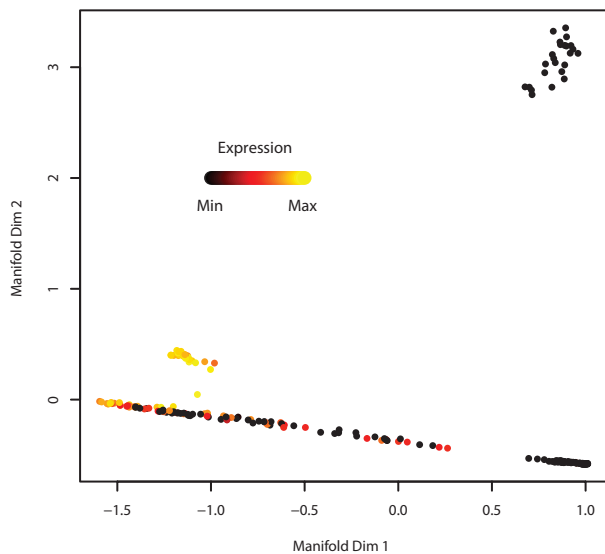**c**

Mbp

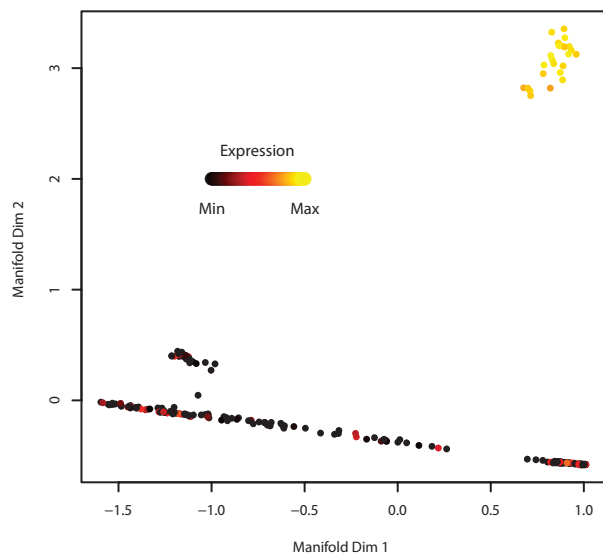**b**

Tubb3

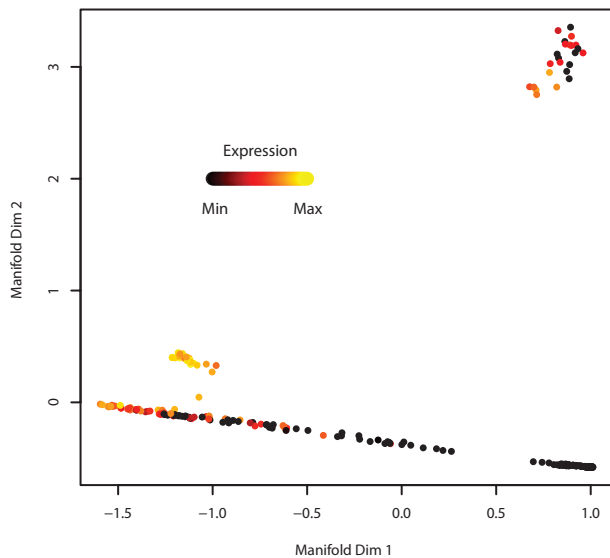**d**

Sv2a

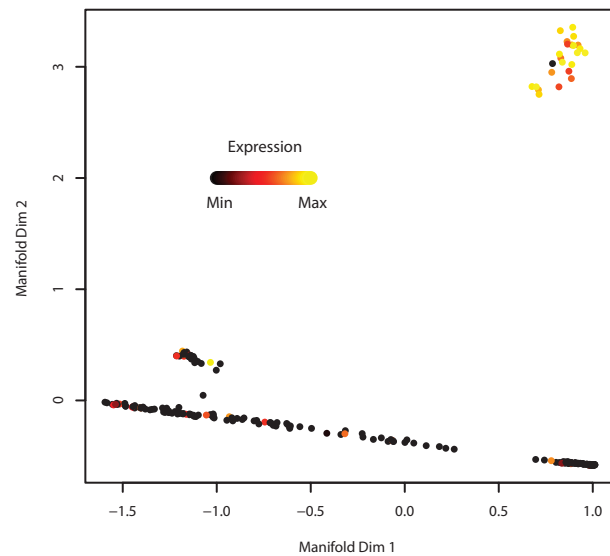

Supplement: Additional file 6: Figure S6. — Additional marker genes for mouse neural stem cell dataset. (a) and (b) are neuroblast markers that also show expression in some active NSCs. (c) and (d) are oligodendrocyte markers. (PDF 100 kb) [file 13059_2016_975_MOESM6_ESM.pdf]

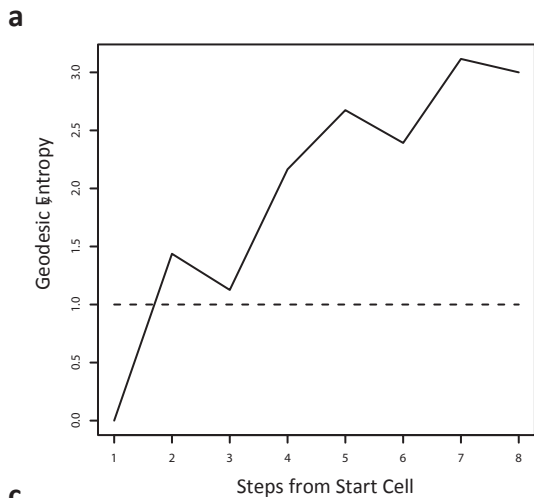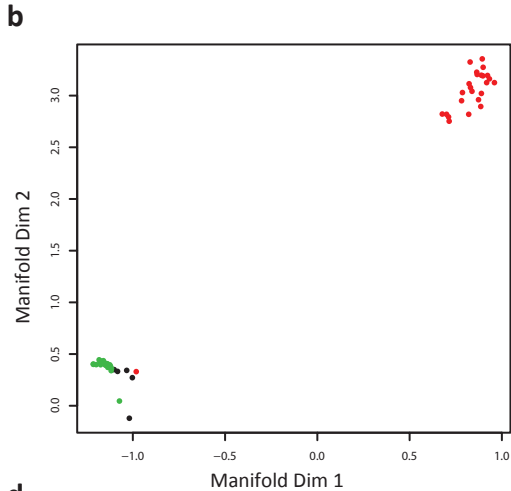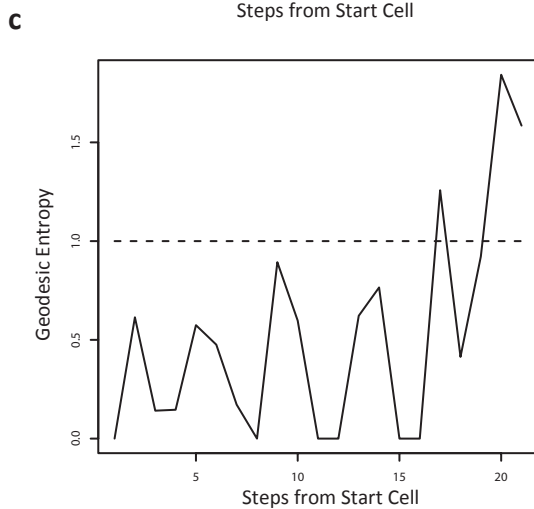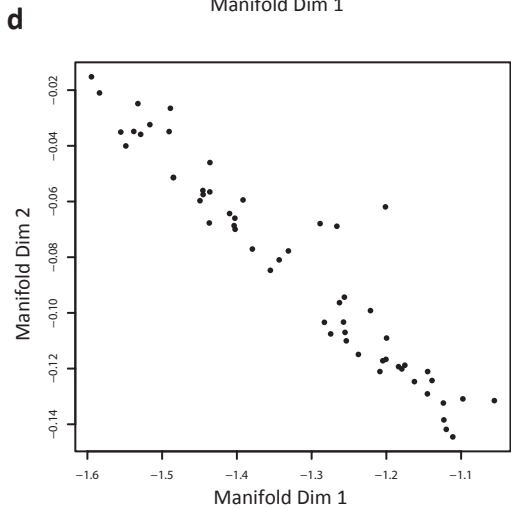

Supplement: Additional file 7: Figure S7. — Detecting multiple branches in the mouse neural stem cell dataset. (a) Geodesic entropy computed recursively for the main trajectory branch containing neuroblasts and oligodendrocytes. Entropy exceeds 1 almost immediately, indicating the presence of a second branch separating neuroblasts and oligodendrocytes. (b) Neuroblasts and oligodendrocyte cells colored by SLICER’s branch assignments. (c) Geodesic entropy computed recursively for the main trajectory branch containing active neural stem cells. Note that geodesic entropy exceeds 1 only near the end of the branch due to the small number of cells at that distance from the starting cell. SLICER does not detect a branch in this case because the number of cells falls below a user-specified threshold (10 by default). (d) Active neural stem cells colored by SLICER’s branch assignments. (PDF 108 kb) [file 13059_2016_975_MOESM7_ESM.pdf]

a

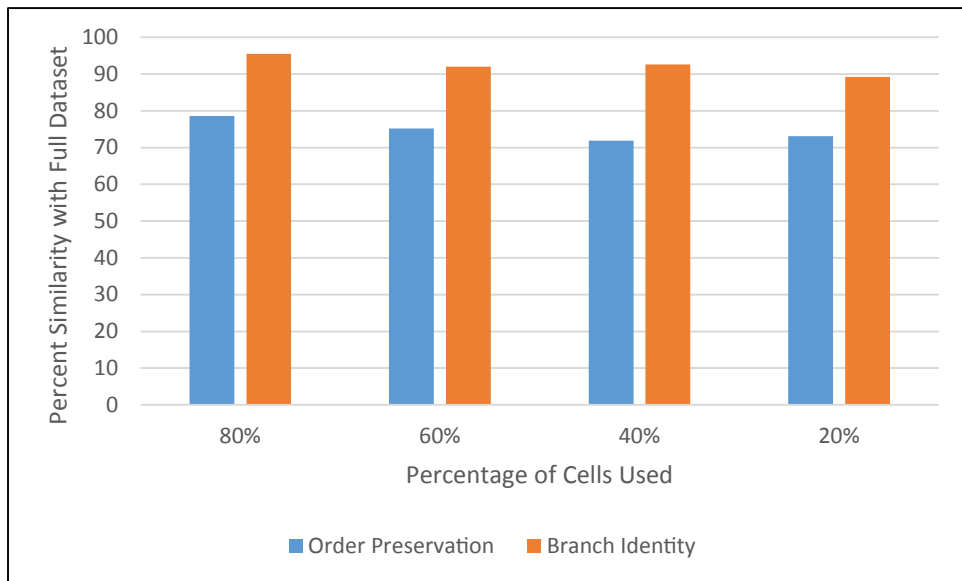

b

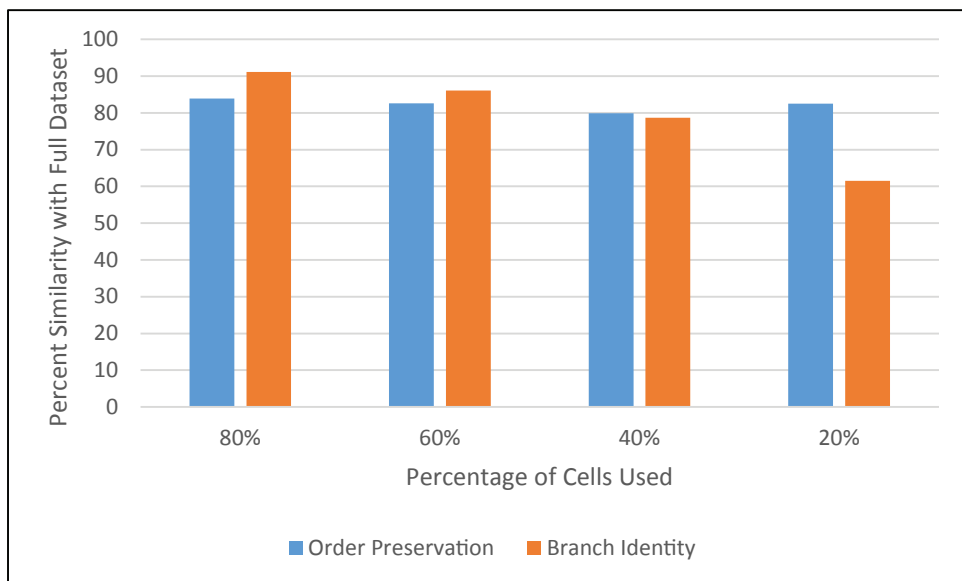

Supplement: Additional file 8: Figure S8. — Accuracy of trajectory reconstruction using a subset of cells. (a) Graph showing how similar the SLICER trajectory is when computed using a random subset of lung cells. The blue bars show the similarity in cell ordering (units are percent sorted with respect to the trajectory constructed from all cells). The orange bars show the similarity in branch assignments (percentage of cells assigned to the same branch as the trajectory constructed from all cells). The values shown were obtained by averaging the results from five subsampled datasets for each percentage (80 %, 60 %, 40 %, and 20 %). (b) Order preservation and branch identity values computed as in panel (a), but for datasets sampled from the neural stem cell dataset. (PDF 106 kb) [file 13059_2016_975_MOESM8_ESM.pdf]

**a**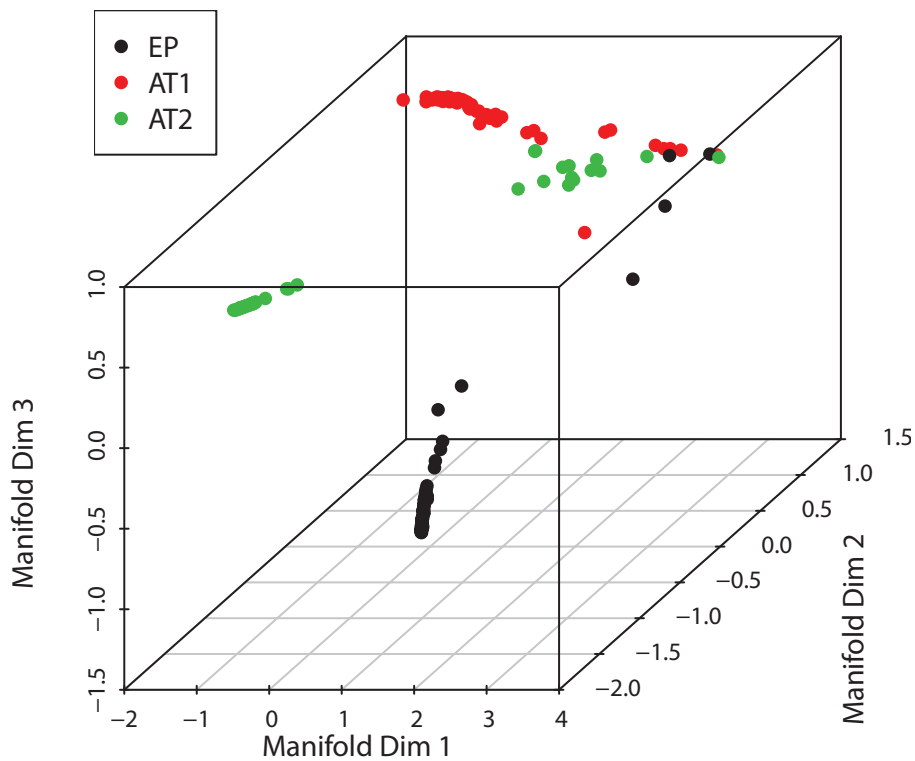**b**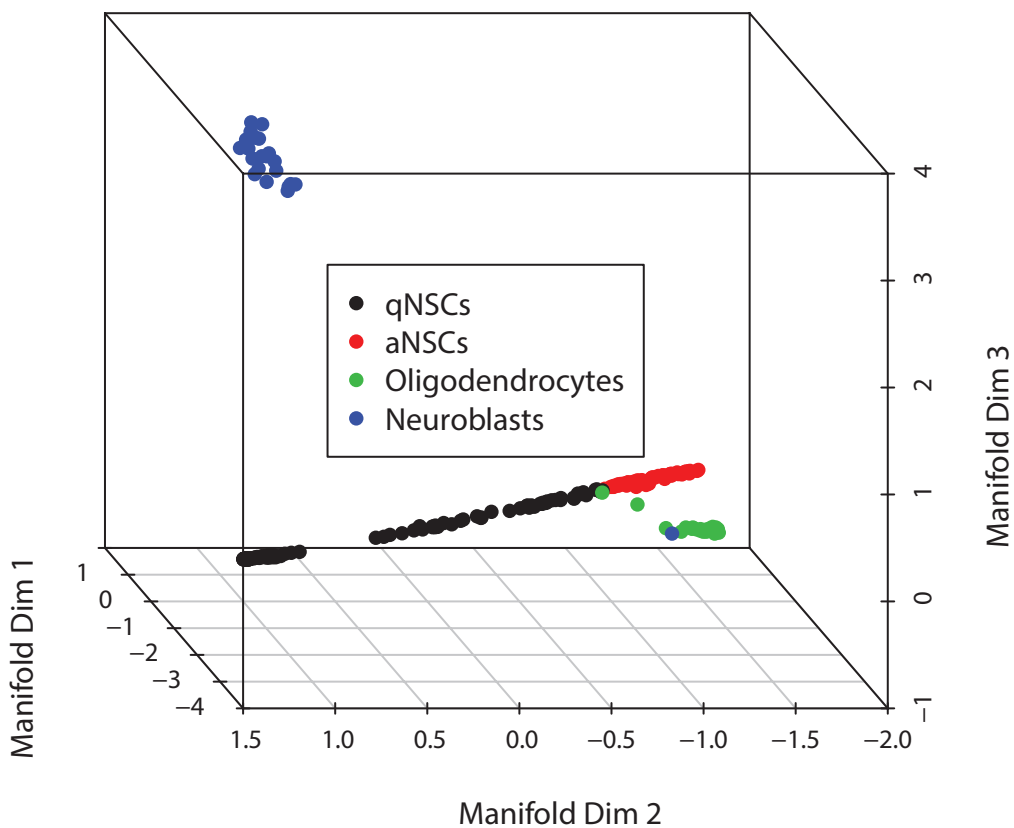

Supplement: Additional file 9: Figure S9. — Three-dimensional LLE results for biological datasets. Points are colored based on SLICER branch assignments using two-dimensional LLE embedding. (a) LLE embedding of distal lung epithelium data. (b) LLE embedding of neural stem cell data. (PDF 87 kb) [file 13059_2016_975_MOESM9_ESM.pdf]

a

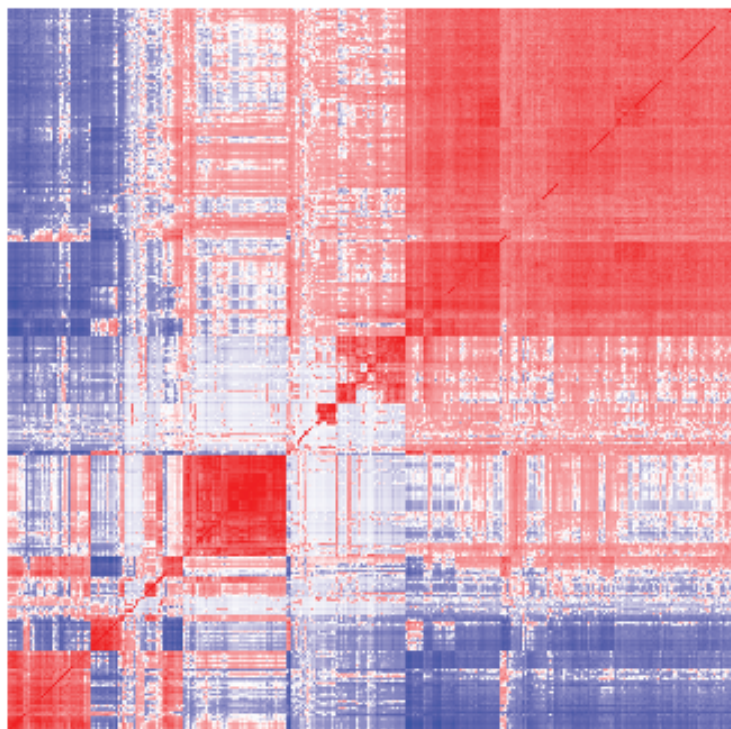

b

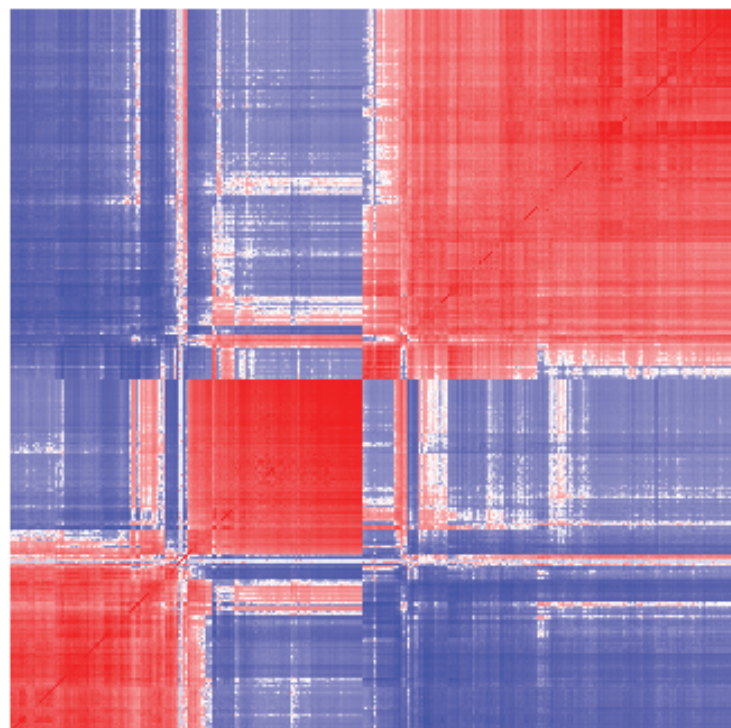

Supplement: Additional file 10: Figure S10. — Correlation matrices for genes selected by SLICER. Blue indicates negative correlation and red indicates positive correlation. (a) Genes selected from the distal lung epithelium dataset. (b) Genes selected from the neural stem cell dataset. (PDF 368 kb) [file 13059_2016_975_MOESM10_ESM.pdf]
